# A FOD Detection Approach on Millimeter-Wave Radar Sensors Based on Optimal VMD and SVDD

**DOI:** 10.3390/s21030997

**Published:** 2021-02-02

**Authors:** Jun Zhong, Xin Gou, Qin Shu, Xing Liu, Qi Zeng

**Affiliations:** School of Electrical Engineering, Sichuan University, Chengdu 610000, China; zhongjun55@163.com (J.Z.); GX199666@163.com (X.G.); liuxing4@126.com (X.L.); zengqi1982@163.com (Q.Z.)

**Keywords:** FOD detection, MMW radar sensor system, SVDD classifier, the optimal VMD

## Abstract

Foreign object debris (FOD) on airport runways can cause serious accidents and huge economic losses. FOD detection systems based on millimeter-wave (MMW) radar sensors have the advantages of higher range resolution and lower power consumption. However, it is difficult for traditional FOD detection methods to detect and distinguish weak signals of targets from strong ground clutter. To solve this problem, this paper proposes a new FOD detection approach based on optimized variational mode decomposition (VMD) and support vector data description (SVDD). This approach utilizes SVDD as a classifier to distinguish FOD signals from clutter signals. More importantly, the VMD optimized by whale optimization algorithm (WOA) is used to improve the accuracy and stability of the classifier. The results from both the simulation and field case show the excellent FOD detection performance of the proposed VMD-SVDD method.

## 1. Introduction

The foreign object debris (FOD) [1] on airport runways, such as screws, animals, stones, plastics and so on, can lead to tragedies of aircraft damage and human mortality. FODs cause severe direct damage and enormous maintenance cost to airport authorities every year around the world. In order to ameliorate this problem, it is urgent to develop a reliable, high-efficiency and automatic FOD monitoring system. At present, the UK Tarsier system [2], the Israeli FODetect system [3], the US FODFinder system [4], and the FOD detection system of Beijing Daxing International Airport in China are in service, which are composed of optical equipment and millimeter-wave (MMW) radar sensor systems. According to the evaluation of the Federal Aviation Administration, the performance of optical instruments is greatly influenced by light, and the detecting probability is very poor in bad weather [5]. On the contrary, millimeter-wave (MMW) radar system is less effected by illumination and climate condition [6], and MMV radar has higher resolution and lower power consumption [7,8,9].

However, the major challenge for radar systems is how to detect weak target signals from the strong ground clutter of airport runway background [10]. The constant false alarm rate (CFAR) methods including cell-average CFAR (CA-CFAR) [11] and clutter-map CFAR (CM-CFAR) [12] are widely utilized in radar automatic target recognition systems. CFAR can automatically adjust the threshold to detect targets when the intensity of external interference changes. But CFAR methods are restricted by the complex background scattering characteristics in airport runway [13] and therefore generate many false alarms.

To solve this problem, some researchers have studied various FOD detection methods. Reference [14] proposed a hierarchical FOD detection scheme based on CM-CFAR and pattern classification. This method adopts hierarchical signal processing mechanism. Firstly, CM-CFAR algorithm is used to process radar echo signals, which reduces the intensity of ground clutter. Then classification method is used to distinguish targets from false alarms, which reduces the false alarm probability and improves the detection probability. Nevertheless, the detection result still depends on the detection effect of CFAR and is influenced by clutter. Reference [15] established a particle swarm optimization-support vector data description (PSO-SVDD) classifier to detect target signals form ground clutter signals that the classifier parameters were optimized by particle swarm optimization (PSO). For PSO-SVDD classifier, a large number of ground clutter signals are used to train the classifier in the training stage. Compared with the traditional CA-CFAR and CM-CFAR method, this method has a better detection performance. However, the classification boundary is easily overfitted due to lack of target sampling data to train the PSO-SVDD classifier. This paper proposes an improved FOD detection approach based on optimal Variational mode decomposition (VMD) and support vector data description (SVDD). The aims of this method are to suppress ground clutter and improve target detection probability.

VMD [16,17], a sparse signal processing method, can decompose a complex signal into an ensemble of band-limited intrinsic mode functions (BLIMFs). The key issue is how to set two crucial parameters of VMD algorithm: the penalty factor α and the decomposition layer number *K*. In [18,19], the VMD parameters are searched by particle swarm optimization (PSO) algorithm and artificial fish swarm algorithm (AFSA), respectively. From their results, it is feasible to apply artificial intelligence (AI) algorithms for VMD parameter optimization. Whale optimization algorithm (WOA) [20] is an efficient search and optimization method in AI algorithms that has many superiorities, such as fewer input parameters, faster convergence speed, and stronger global search ability. SVDD [21] is one of the most prominent methods for one-class classification that is to find a set containing the most typic instances and rejecting abnormal instances. In this paper, the parameters of VMD are first optimized by WOA. Then radar echo signals are decomposed into FOD intrinsic mode functions (IMFs) and clutter IMFs with the optimal VMD. Finally, clutter IMFs are utilized to train the SVDD classifier, and then targets in FOD IMFs are detected by the trained classifier. Here, the optimal VMD algorithm can not only improve the classification effect of the SVDD classifier but also ensure the stable working performance of the classifier. Experiments are conducted to validate that the proposed VMD-SVDD method can yield preferable detection results in simulation and practical measure.

The rest of this paper is organized as follows. In Section 2, the distance measuring principle of linear frequency modulated continuous wave (LFMCW) radar is introduced. The proposed method is described in detail in Section 3. The Section 4 gives the experimental processes and results. The conclusions are drawn in Section 5.

## 2. Distance Measuring Principle of LFMCW Radar

Linear frequency modulated continuous wave (LFMCW) [22] radar is frequently used in radar sensors on airport runways. It is beneficial to detect weak targets under strong ground clutter with LFMCW radar due to the advantages of high range resolution and low transmitting power.

**Figure 1:**
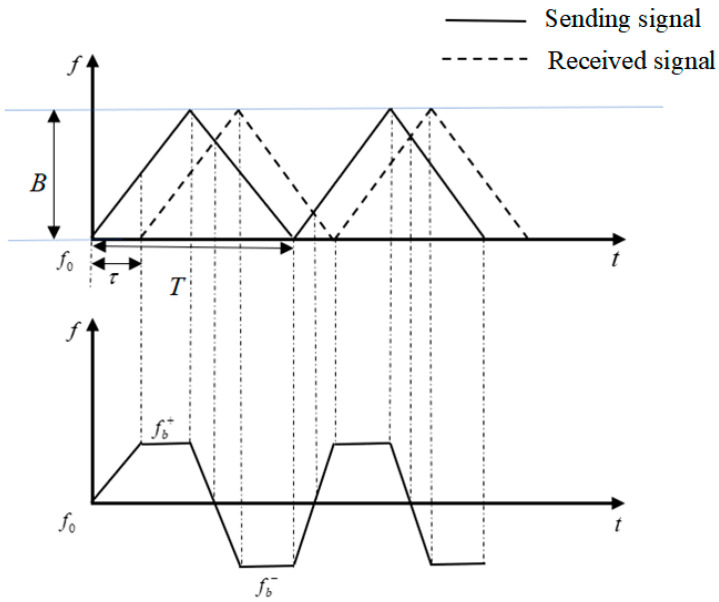
Time-frequency diagram of LFMCW radar.

Figure 1 is the time-frequency diagram of LFMCW radar. The factors *B*, *T*, *f_T_*, *f_R_*, and *f*_0_ represent the band-width, modulation cycle, sending frequency, receive frequency and center frequency. $f_{b+}$ and $f_{b-}$ are positive beat frequency and negative beat frequency. *τ* is time delay. The ranging principle of LFMCW radar is as follows.

The frequency of the sending signal is:

(1) $$f_{T}=f_{0}+\frac{Bt}{T}$$

Suppose the target distance is R. The frequency of the received signal is:

(2) $$f_{R}=f_{0}+\frac{B}{T}(t-\frac{2R}{c})$$

The light speed is *c*. The signal delay is τ=2R/c. The beat frequency is:

(3) $$f_{b}=f_{R}-f_{T}=\frac{2RB}{cT}$$

$f_{b}$ can be calculated by spectrum analysis, and then the target distance *R* can be obtained.

In radar sensors, radar echo signals are mixed with clutter and noise. The noise is mainly the thermal noise in radar receivers, which is much weaker than ground clutter, so it is ignored in this paper. The final received signal can be formulated as:

(4) $$s_{R}(t)=s_{fod}(t)+s_{clutter}(t)$$

where, $s_{fod}\left(t\right)$ is the target signal, and $s_{clutter}\left(t\right)$ is the clutter signal. Supposing the sending signal is $s_{T}(t)$, the signal through the mixer receiver can be expressed as

(5) $$s(t)=s_{R}(t)\times s_{T}(t)$$

Based on fast Fourier transform (FFT), it is easy to get the target distance in frequency domain.

## 3. Proposed Method and Explanation

**Figure 2:**
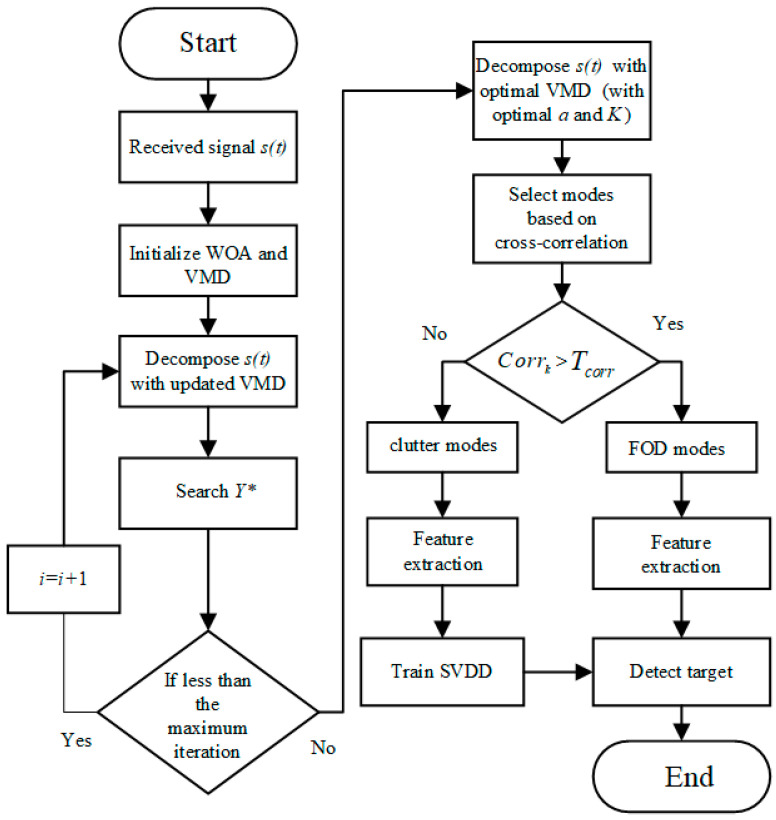
Flowchart of the proposed method.

The flowchart of the proposed VMD-SVDD method is shown in Figure 2. It is described subsequently in detail.

### 3.1. VMD Parameter Optimization

Variational mode decomposition (VMD) is one of the sparse signal processing methods, which was proposed in 2014. The VMD can automatically and effectively decompose a complex signal into an ensemble of band-limited intrinsic mode functions (BLIMFs). VMD can be used for filtering and denoising. Compared with other signal decompose methods such as empirical modal decomposition (EMD) [23] and ensemble empirical mode decomposition (EEMD) [24], VMD overcomes problems of modal aliasing, end-point effect, and false component.

The VMD is used to decompose the mixing signal s(t) into a finite number of BLIMFs. The BLIMF has a central frequency and a limited bandwidth. The constrained variational model is constructed as follows:

(6) $$\left\{ \begin{array}{l} \min_{\left\{h_{k}\right\},\left\{w_{k}\right\}}\left\{\sum_{K}{\left\|\partial_{t}\left[\left(\delta (t)+j\frac{1}{\pi t}\right)*h_{k}(t)\right]e^{-jw_{k}t}\right\|}_{2}^{2}\right\}\\ s.t.\sum_{k=1}^{K}h_{k}=s(t) \end{array}\right.$$

where $\left\{h_{k}\right\}=\left\{h_{1},h_{2},h_{3},\ldots ,h_{K}\right\}$ is intrinsic mode function, $\omega_{k}=\left\{\omega_{1},\omega_{2},\omega_{3},\ldots ,\omega_{K}\right\}$ is the center frequency of $h_{k}$, δ(t) is the unit impulse function, and $\partial_{t}$ is the gradient with respect to the time series.

To solve the constrained variation problem, quadratic penalty parameter α and Lagrange operator λ [25] are introduced. The extended Lagrange expression is given as follows:

(7) $$L\left(\{h_{k}\},\{w_{k}\},\lambda \right)=\alpha *\sum_{K}{\left\|\partial_{t}\left[\left(\delta (t)+\frac{j}{\pi t}\right)*h_{k}(t)\right]e^{-jw_{k}t}\right\|}_{2}^{2}+{\left\|s(t)-\sum_{K}h_{k}(t)\right\|}_{2}^{2}+\langle \lambda (t),s(t)-\sum_{K}h_{k}(t)\rangle$$

By using the alternating direction multiplicative operator method (ADMM) [26], $h_{k}^{n+1}$, $w_{k}^{n+1}$, and $\lambda^{n+1}$ are updated until the stop condition is satisfied. Among:

(8) $$h_{k}^{n+1}(t)=\underset{h_{k}\in s}{\arg\min}\{\alpha *{\left\|\partial_{t}\left[\left(\delta (t)+\frac{j}{\pi t}\right)*h_{k}(t)\right]e^{-jw_{k}t}\right\|}_{2}^{2}+{\left\|s(t)-\sum_{K}h_{k}(t)+\frac{\lambda (t)}{2}\right\|}_{2}^{2}\}$$

In the VMD processing, penalty factor α and decomposition layer number *K* need to be known in advance. The integrity of the decomposed component is determined by α. An improper *K* may lead to many problems, such as mode aliasing and false components. The whale optimization algorithm (WOA) is selected to search suitable α and K for the VMD in this paper.

Whale optimization algorithm (WOA) is a new heuristic optimization algorithm of artificial intelligence algorithms, which has advantages of fewer input parameters, faster convergence speed, and stronger global search ability. The WOA algorithm simulates hunting behaviors of humpback whales. The mathematical model of WOA can be expressed as:

In a given space, whales will constantly update their positions to get close to the prey position. That is to say: Give a range of $\alpha \in \left[\alpha_{1},\alpha_{n}\right]$ and $K\in \left[K_{1},K_{m}\right]$. ${\vec{Y}}_{i}$ and ${\vec{Y}}^{*}$ are the whale positions and the prey position respectively. ${\vec{Y}}_{i}=(\alpha_{f},K_{g})$, 1 ≤ *f* ≤ *n*, 1 ≤ *g* ≤ *m*, 1 ≤ *i* ≤ *nm*, ${\vec{Y}}^{*}$ is first given at random. ${\vec{Y}}_{i}$ will keep updating to get closer to ${\vec{Y}}^{*}$. At the same time, ${\vec{Y}}^{*}$ is kept or updated to the optimal position in each iteration.

There are two whales foraging behaviors, namely, shrinking encircling mechanism and spiral position updating. The probability of both behaviors is 50%. The model is given as follows:

(9) $${\vec{Y}}_{i+1}=\left\{ \begin{array}{cc} {\vec{Y}}_{i}^{*}-\vec{A}\cdot \vec{D} & p<0.5\\ {\vec{D}}^{*}\cdot e^{bl}\cdot \cos(2\pi l)+{\vec{Y}}_{i}^{*} & p\geq 0.5 \end{array}\right.$$

When p<0.5:

(10) $$\vec{D}=\left|\vec{C}\cdot {\vec{Y}}_{i}^{*}-{\vec{Y}}_{i}\right|$$

(11) $${\vec{Y}}_{i+1}={\vec{Y}}_{i}^{*}-\vec{A}\cdot \vec{D}$$

Among, *i* is the number of iterations, which is restricted to less than the maximum number of iterations. $\vec{A}=2\cdot \vec{a}\cdot \vec{d}-\vec{a}$ and $\vec{C}=2\cdot \vec{d}$ are coefficient vectors, $\vec{a}$ is linear regressive from 2 to 0, and $\vec{d}$ is a random vector in [0,1].

When p≥0.5:

(12) $${\vec{D}}^{*}=\left|{\vec{Y}}_{i}^{*}-{\vec{Y}}_{i}\right|$$

(13) $${\vec{Y}}_{i+1}={\vec{D}}^{*}\cdot e^{bl}\cdot \cos(2\pi l)+{\vec{Y}}_{i}^{*}$$

where *b* is a constant which determines the logarithmic spiral shape, and *l* is a random number in [−1,1].

In order to strengthen global search ability of WOA, whales also randomly search for the prey. If $\vec{A}\geq 1$, ${\vec{Y}}_{i+1}$ will be updated according to a random whale position ${\vec{Y}}_{rand}$. The mathematical model is:

(14) $$\vec{D}=\left|\vec{C}\cdot {\vec{Y}}_{rand}-{\vec{Y}}_{i}\right|$$

(15) $${\vec{Y}}_{i+1}={\vec{Y}}_{rand}-\vec{A}\cdot \vec{D}$$

During each iteration, the prey position ${\vec{Y}}^{*}$ always corresponds to the current maximum kurtosis of cross-correlation coefficients between s(t) and $h_{k}(t)$. In the end, ${\vec{Y}}^{*}$ is consisted of optimal α and K. Then s(t) is decomposed by VMD with optimal parameters. Select FOD IMFs and clutter IMFs by the average of cross-correlation coefficients between s(t) and $h_{k}(t)$.

### 3.2. SVDD Classification

Support vector data description (SVDD) [27] algorithm is one of the machine learning algorithms, based on traditional support vector machines (SVM) [28,29]. The SVDD has been widely applied in anomaly detection, fault detection and diagnosis process. The basic principle of SVDD is that a minimum hypersphere containing all training samples is generated in the high-dimensional space. The support vectors are the sample points on the surface of the hypersphere. Test samples are normal if them are in the hypersphere, otherwise them are abnormal samples. The training samples (clutter signals in this paper) are $X_{t}=\left\{x_{t_{1}},x_{t_{2}},\ldots ,x_{t_{N}}\right\}$. The optimization problem of SVDD can be expressed as the follows:

(16) $$\begin{array}{l} \min B(c,r^{\prime })={r^{\prime }}^{2}+C\sum_{i=1}^{N}\xi_{i}\\ s.t.{\left(x_{t_{i}}-c\right)}^{T}(x_{t_{i}}-c)\leq {r^{\prime }}^{2}+\xi_{i}\\ \xi_{i}\geq 0,\forall_{i}=1,\ldots ,N \end{array}\}$$

where $C\sum_{i=1}^{N}\xi_{i}$ is used to reduce the influence of outliers. $\xi_{i}$ is the slack variable, and every data point has a corresponding one. When two data points have the same the slack variable, the hyperspheres are the same. C determines the decision boundary of training samples. $r^{\prime }$ is the radius. c is the sphere center. The hypersphere can be obtained by using kernel function [30] and Lagrange multipliers. This paper uses the popular Gaussian kernel function, which is defined as:

(17) $$K(x_{t_{i}},x_{t_{j}})=\exp(\frac{-{\left\|x_{t_{i}}-x_{t_{j}}\right\|}^{2}}{\sigma^{2}})$$

where δ is a parameter that is used to control tightness of the boundary. Gaussian kernel function provides an efficient technique to map the data of the input space into the high-dimensional feature space. The dual form of the Equation (16) guided by the Gaussian kernel function and Lagrange multipliers is:

(18) $$\begin{array}{l} \max L=\sum_{i=1}^{n}{\alpha^{\prime }}_{i}K(x_{t_{i}},x_{t_{j}})-\sum_{i,j}{\alpha^{\prime }}_{i}{\alpha^{\prime }}_{j}K(x_{t_{i}},x_{t_{j}})\\ s.t.\sum_{i=1}^{n}{\alpha^{\prime }}_{i}=1\\ 0\leq {\alpha^{\prime }}_{i}\leq C,\forall_{i}=1,\ldots ,N \end{array}\}$$

where ${\alpha^{\prime }}_{i}$ corresponds to $x_{t_{i}}$. when ${\alpha^{\prime }}_{i}>0$, $x_{t_{i}}$ is the support vector. Then the sphere center *c* and the radius $r^{\prime }$ can be obtained. The hypersphere is inversely mapped to the original data space to get the decision boundary of the FOD and clutter.

Before SVDD classification, it is necessary to extract two-dimensional features of the FOD and clutter as inputs of the classifier. The autocorrelation distribution of FOD signals is more concentrated while that of ground clutter is more dispersive on account of target echo signals have stronger correlation than clutter signal. The second-order center distance (F1) and fourth-order cumulant (F2) of normalized autocorrelation are chosen as two-dimensional features, which can increase the difference between FODs and clutter. F1 represents the distribution of normalized autocorrelation relative to the geometric center of mass. The more dispersed of the distribution is, the smaller F1 is. F2 can further solve the problems that cannot be solved by F1, such as Gaussian noise. The calculation is as follows:

The normalized autocorrelation coefficient of $h_{k}$ is $r_{i}=\left\{r_{1},r_{2},\ldots ,r_{K}\right\}$,

(19) $$r_{i}=\frac{\sum_{m=1}^{M-h}\left(r_{i_{m}}-{\tilde{r}}_{i})(r_{i_{m+h}}-{\tilde{r}}_{i}\right)}{\sum_{m=1}^{M}{\left(r_{i_{m}}-{\tilde{r}}_{i}\right)}^{2}}$$

where M is the length of $r_{i_{m}}$. 0≤h≤M. 0≤i≤K. ${\tilde{r}}_{i}$ is the average of $r_{i_{m}}$.

Feature 1: the second order center distance of the normalized autocorrelation coefficient

(20) $$F1=\sum_{m=1}^{M}{\left(m-\tilde{m}\right)}^{2}r_{i_{m}}$$

where $\tilde{m}=\sum_{m=1}^{M}{m*p}_{m}$, $p_{m}=r_{i_{m}}/\sum_{m=1}^{M}r_{i_{m}}$.

Feature 2: fourth-order cumulant of normalized autocorrelation coefficient

(21) $$F2=\sum_{m=1}^{M}{\left(m-\tilde{m}\right)}^{4}r_{i_{m}}-{3[\sum_{m=1}^{M}{\left(m-\tilde{m}\right)}^{2}r_{i_{m}}]}^{2}$$

## 4. Discussion

The operation and signal processing parameters of the radar system are provided in Table 1. The carrier frequency is 96 GHz, and the modulation signal is the linear frequency modulated signal with the bandwidth of 1.5 GHz. The polarization is VV. Radar antenna beam width should be as small as possible to obtain better resolution of transverse range. In this paper, the horizontal beam width is 1.9°, the pitch beam width is 5°, and the range resolution is 0.1 ms. The angular step is 12°/s, so the maximum pulse accumulation time is calculated to be 158 ms. The paper adopts the cumulative time of 60 ms, according to the actual target detection strategy and the transmission between upper computer and radar sensor.

### 4.1. Signal Decomposition and Mode Selection

**Figure 3:**
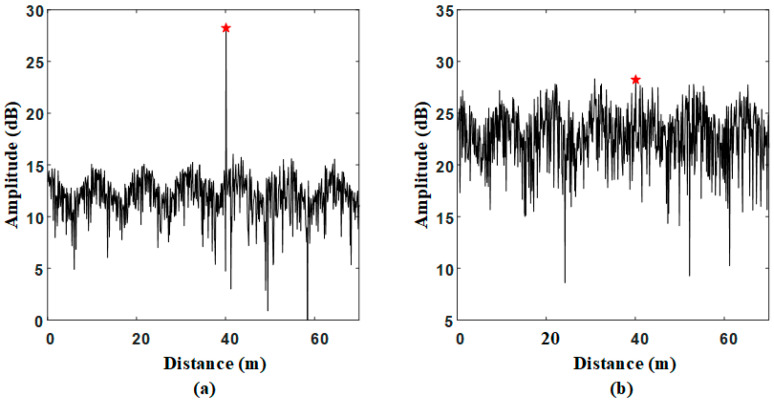
Simulation data: (**a**) Target signals without clutter; (**b**) Target signals with clutter.

In the simulation, the ground clutter signal is the signal whose amplitude distribution satisfies Rayleigh distribution, and the target signal is linear frequency modulation signal. Suppose that there is a target at a distance of 40 m, which is marked with red star in the following figures. Without clutter signals, the target signal is obvious after frequency mixing and fast Fourier transform (FFT) processing that shown in Figure 3a. When the signal-to-clutter rate (SCR) is −20 dB, the target signal almost hides in clutter signals in Figure 3b. It can be seen that the target at 40 m is obscured by the surrounding strong clutter in the amplitude spectrum. At this point, it is difficult to detect the target with the ordinary CFAR method.

**Figure 4:**
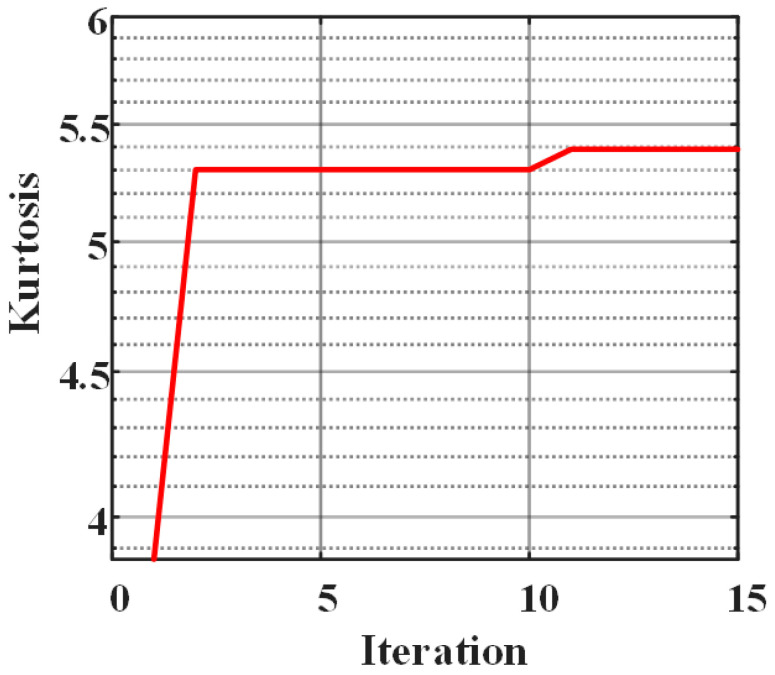
Optimization process of the WOA.

In the proposed method, firstly, quadratic penalty parameter α and decomposition layer number K of variational mode decomposition (VMD) need to be searched by whale optimization algorithm (WOA). In the experiment, the initial ranges of α and K are [1000, 2000] and [5, 10]. The fitness function (objective function) is the maximum kurtosis of cross-correlation coefficients between s(t) and $h_{k}(t)$ of the updating VMD. The maximum iteration is 15. The parameter optimization process of WOA is shown in Figure 4. The maximum kurtosis appears after 11 iterations. At this time, the optimal parameters searched by the WOA are *K* = 7.69 and α = 1497.10, and the integer formats of them are *K* = 7 and α = 1500, which are provided for the VMD.

**Figure 5:**
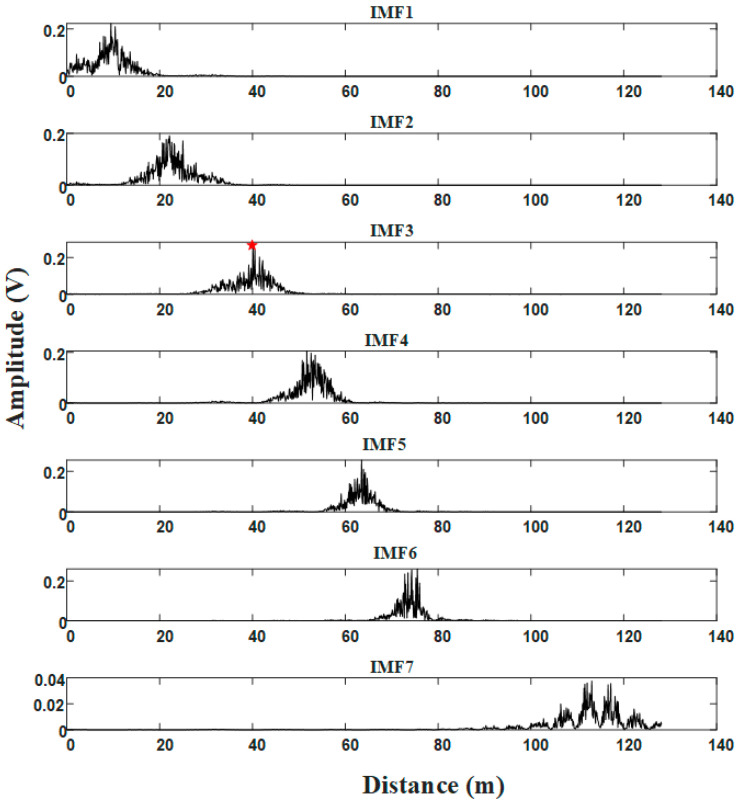
Signal decomposition using the optimal VMD.

Then, the signals in Figure 3b are decomposed by the optimal VMD. The result is shown in Figure 5. These modes are distributed from low frequency to high frequency. The target is assigned to the IMF3, and clutter signals are divided into other IMFs. It can be seen form the IMF3 that the clutter signals around the target is reduced.

To select FOD mode component, set a selection threshold which is the average of cross-correlation coefficients of s(t) and $h_{k}(t)$. The first IMF has no value by analyzing measured data and the last IMF is the residual, these two modes are discarded in the threshold estimation. The threshold value is calculated as follows:

(22) $$Corr_{k}=\frac{\sum_{i=1}^{n}\left(s_{i}-\tilde{s})(h_{k_{i}}-{\tilde{h}}_{k}\right)}{\sqrt{\sum_{i=1}^{n}{\left(s_{i}-\tilde{s}\right)}^{2}\sum_{i=1}^{n}{\left(h_{k_{i}}-{\tilde{h}}_{k}\right)}^{2}}},k=2,3,\ldots ,K-1$$

(23) $$T_{corr}=\frac{1}{5}(Corr_{2}+Corr_{3}+\ldots +Corr_{K-1})$$

n is the length of s(t) and $h_{k}(t)$. $\tilde{s}$ is the average of s(t). ${\tilde{h}}_{k}$ is the average of $h_{k}(t)$.

**Figure 6:**
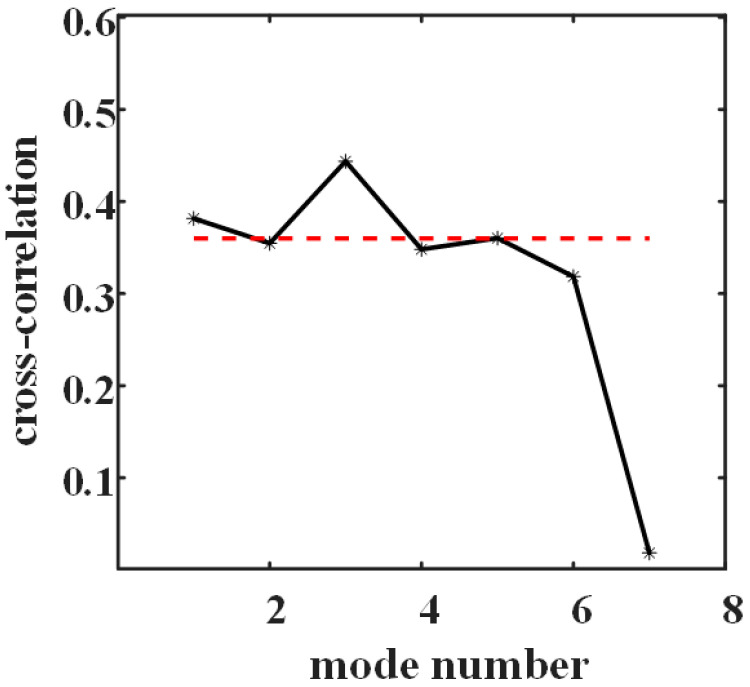
IMF selection with the cross-correlation threshold.

Figure 6 shows the mode selection result of modes in Figure 5, and the red dotted line is the threshold. The mode whose cross-correlation coefficient exceeds the threshold is selected as FOD mode component; otherwise, it is classified as clutter mode component. The cross-correlation coefficient of the mode component including targets is bigger due to target echo signals have stronger correlation than clutter signals. Here, IMF3 and IMF5 are FOD mode components, and IMF2, IMF4 and IMF6 are clutter mode components. The IMF1 includes strong clutter signals near radar sensor, which will improve the threshold value of mode selection. So, it will be discarded in the later signal processing.

In addition, the following experiments have been conducted to illustrate the importance of setting appropriate VMD parameters and the cross-correlation threshold.

**Figure 7:**
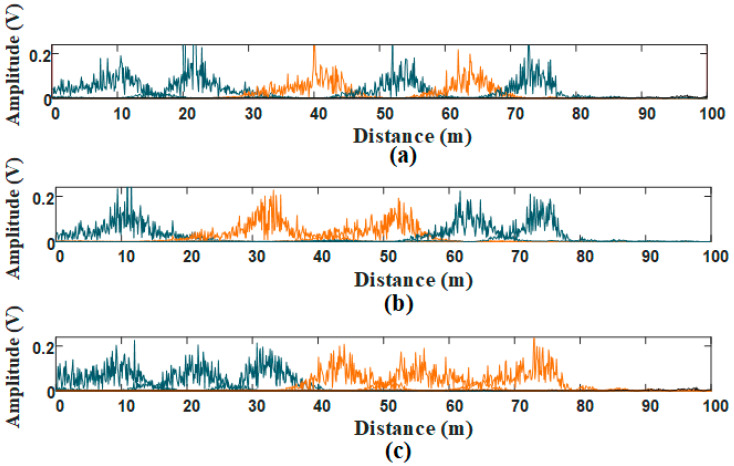
VDM processing with different parameters: (**a**) VMD parameters are α=2000 and K=7; (**b**) VMD parameters are α=2000 and K=5; (**c**) VMD parameters are α=1000 and K=7.

The simulation data in Figure 3b is decomposed by VMD with different penalty factor α and decomposition layer number K. These mode components are shown in Figure 7. The yellow parts are selected as target modes by the cross-correlation threshold. Compared with the results in Figure 7b,c, the target signal at 40 m in Figure 7a is intact when VMD parameters are α = 2000 and *K* = 7. In Figure 7b, α = 2000 and *K* = 5. The inaccurate decomposition is caused by improper K, bringing false components. In Figure 7c, α = 1000 and *K* = 7. The integrity of target signal is destroyed by the inappropriate α. In Figure 7b,c, the target signals in selected target modes are weaken, which are almost undetectable in the next signal processing. Therefore, it is necessary to optimize VMD parameters by WOA, which ensures the integrity of target signals.

**Figure 8:**
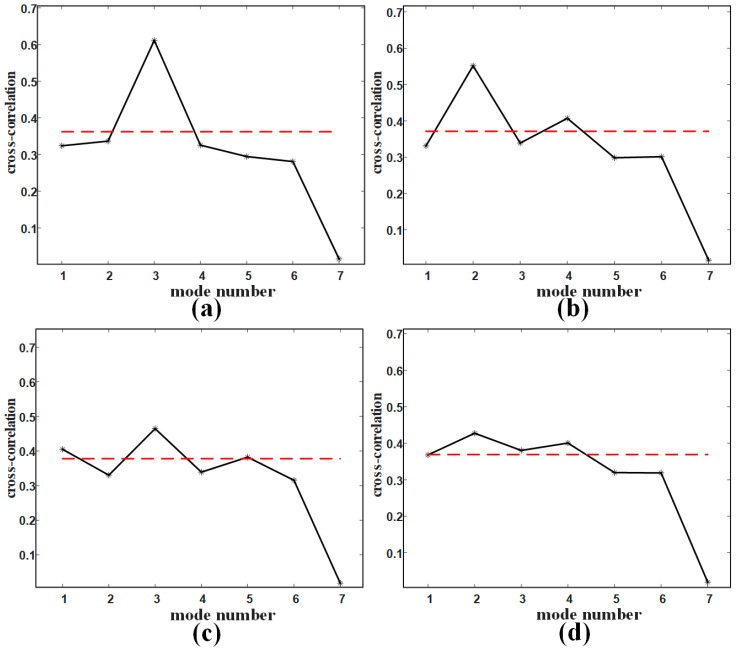
Mode selection results in four cases: (**a**) Single target with SCR=−15 dB; (**b**) Two targets with SCR=−15 dB; (**c**) Single target with SCR=−20 dB; (**d**) Two targets with SCR=−20 dB.

Figure 8 shows the results of mode selection based on the the cross-correlation threshold in four cases. There is a target 40 m form the radar sensor in Figure 8a,c. And there are two targets in Figure 8b,d: one is 25 m from the radar sensor, the other is 55 m from the radar sensor. The SCR is −15 dB in Figure 8a,b, and the SCR is −20 dB in Figure 8c,d. In Figure 8, the abscissa is the serial number of the mode, the y-coordinate is the cross-correlation coefficient, and the red dotted line is the threshold.

It can be seen from these selection results that, with the decrease of signal-to-clutter rate (SCR), the number of modes below the threshold decreases and the selected clutter modes decrease, regardless of single target or multiple targets. From the above simulation results, the number of clutter data used to train the classifier is sufficient, although some of clutter modes are selected as target modes. Moreover, if the number of targets increases further, more clutter modes can be obtained by increasing the decomposition layer of VMD. But this case of too many targets on airport runways is not common.

### 4.2. SVDD Classification and Fod Detection

After the mode components are divided into FOD modes and clutter modes by the cross-correlation threshold, the SVDD classifier is used to distinguish FOD signals from clutter signals.

In the simulation, two important parameters of SVDD are set as follows: *δ* = 0.5 and *C* = 1, which are optimized by particle swarm optimization (PSO) [15]. According to Equations (20) and (21), feature vectors of the training samples (clutter signals) are obtained to train SVDD classifier.

**Figure 9:**
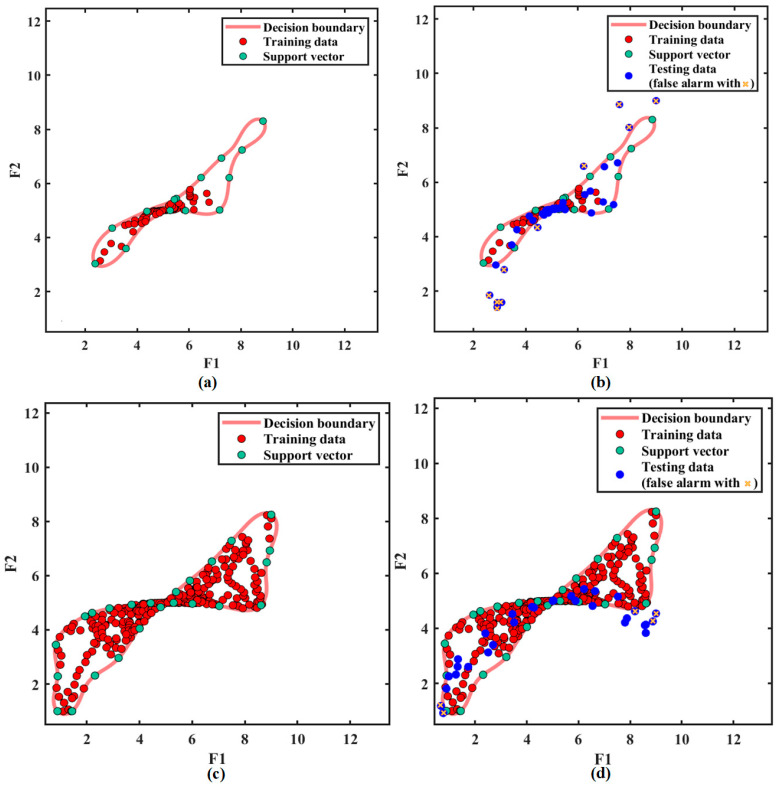
Classification results: (**a**) Training result without VMD processing; (**b**) Testing result without VMD processing; (**c**) Training result with VMD processing; (**d**) Testing result with VMD processing.

Without VMD processing, after the SVDD classifier is trained by clutter signals in the Figure 3b, the red optimal decision boundary is obtained as shown in Figure 9a. In the training stage, the optimal decision boundary is obtained. In the testing stage, if sampling points falls within the boundary, they are considered as clutter signals. Otherwise, they will be considered as non-clutter signals. The simulation data from Figure 3b are tested by the trained SVDD classifier. The classification result is shown in Figure 9b. The sampling points outside the boundary are false alarms, which are marked with yellow cross in figures. The sampling points of the target can hardly be found due to the effect of strong clutter.

With VMD processing, the clutter modes in Figure 5 are selected to train the classifier and the FOD modes are tested by the trained VMD-SVDD classifier. Figure 9c shows the training result and Figure 9d shows the testing result. Although there are still some false alarms outside the decision boundary, some sampling points of the target can be detected. Here, a target corresponds to multiple sampling points in the figure due to radar signals are linear frequency modulated continuous wave signals. By Comparing the results in Figure 9b,d, it is easier to detect targets in Figure 9d under the same SCR. As can be seen from above experiments, VMD processing can reduce the effect of clutter on target signals, which improves the SVDD classification efficiency.

**Figure 10:**
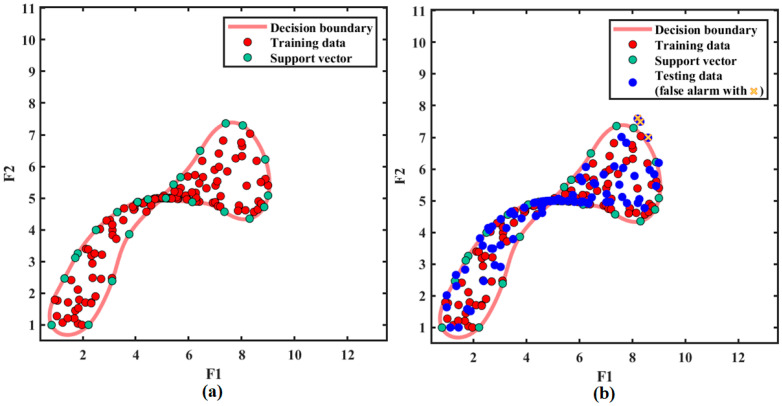
Classification results of clutter data set: (**a**) Training result; (**b**) Testing result.

In order to test the classification performance of VMD-SVDD classifier, the proposed method is tested on a clutter data set without targets. In this case, the cross-correlation mode selection algorithm will mistakenly select some of the clutter modes as the target modes (called false target modes). However, sampling points of these false target modes are likely to be classified into the decision boundary by the trained classifier. As can be seen from Figure 10, false alarms appear very close to the decision boundary. After a large number of repeated experiments, it is found that the false alarm probability of the proposed method is about 0.8%.

For the training of VMD-SVDD classifier, the training set includes clutter modes selected by the cross-correlation threshold after VMD processing. While, the testing set includes target modes, and may also include other clutter modes. From above simulation and test results, it can be seen that using clutter modes as a test set results in an error probability of about 0.8%, which has little effect on target detection. So, the training way of the VMD-SVDD classifier proposed in this paper is reliable.

To compare the detection performance of the proposed VMD-SVDD method, four methods are applied to detect a same target under different SCR. The cell-average CFAR (CA-CFAR) method is to obtain a detection threshold by averaging the clutter power around the target. If the amplitude of a signal exceeds the threshold, the signal is considered as a target. The principle of VMD-CACFAR method is to detect targets in FOD mode components by CA-CFAR method. In SVDD method and the proposed VMD-SVDD method, target sampling points are marked in advance, and the target is detected by confirming that sampling points outside the decision boundary are labeled.

**Figure 11:**
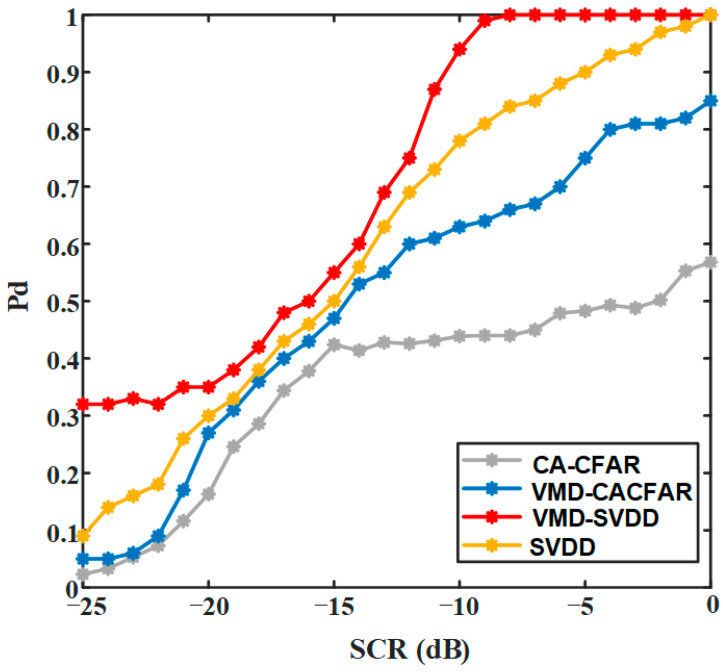
Detection probabilities of four detection methods.

The numbers of simulation are 1000 in every case and the detection probability ($P_{d}$) result is shown in Figure 11. Under different SCR, $P_{d}$ of the proposed VMD-SVDD method is higher than that of other methods. Especially, the target can almost always be accurately detected when *SCR* > −10 dB. It can be seen from Figure 10 that the detection performance based on SVDD or VMD-SVDD is better than that based on CA-CFAR or VMD-CACFAR. Compared with the SVDD classifier, the proposed VMD-SVDD classifier has the higher detection probability, which proves that the optimal VMD can improve the accuracy of SVDD classification.

Then the real-time analysis of above four methods is carried out, and the time required to run these algorithms is shown in Table 2. Among them, the running time of the proposed algorithm is 10.93 s, which is acceptable in actual projects. By comparing the running time and detection probability of above four detection methods, it can be seen that while the detection probability of the proposed method is high, the running time is long. In particular, VMD algorithm increased running time. In future work, the VMD module can be optimized to improve the run time of the proposed method.

### 4.3. Field Measure and Validation Result

**Figure 12:**
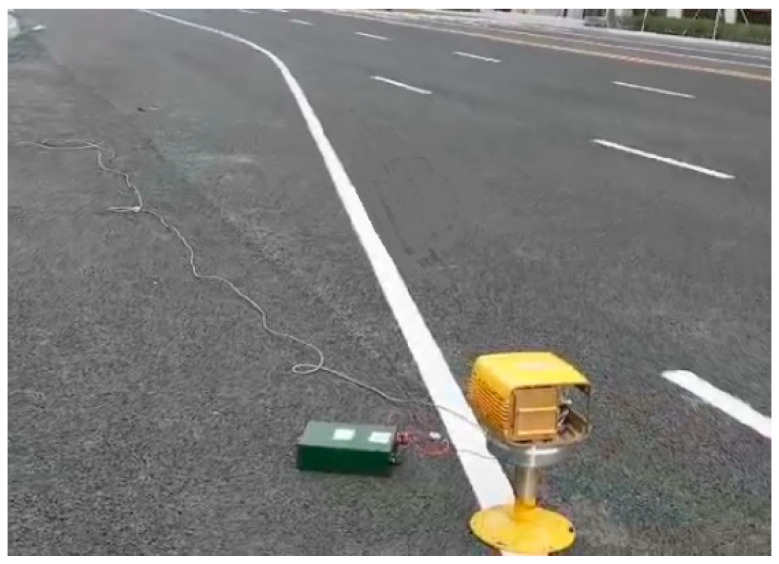
Field measure scenario.

To verify general validity of the proposed VMD-SVDD method, it is applied to a radar sensor system in reality. Figure 12 is the photograph of field measure scenario. Two cases were tested in this field: in scene 1, a metal ball was placed 40 m from the radar sensor; in scene 2, two bolts were placed 40 m and 55 m from the radar sensor. The radar echo signals are processed by the proposed VMD-SVDD method.

**Figure 13:**
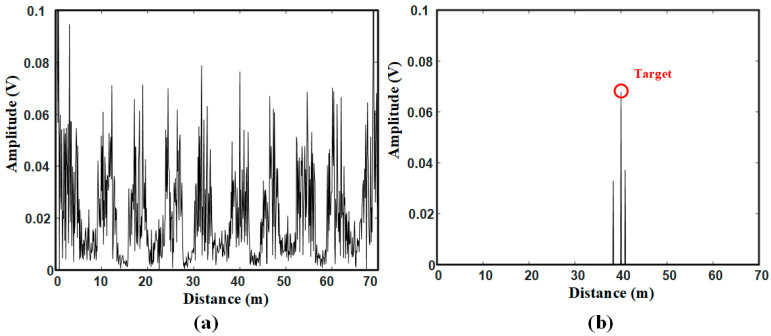
Signals in scene 1: (**a**) Original signal; (**b**) Target detection result.

For scene 1, the original signal after frequency mixing and FFT processing is shown in Figure 13a. As can be seen that the signal of the metal ball 40 m from the radar sensor is obscured by surrounding strong clutter signals. After the proposed method processing, the detection result is shown in Figure 13b that the target signal can be detected with two false alarms existing around the target. The problem of false alarms will be discussed later.

**Figure 14:**
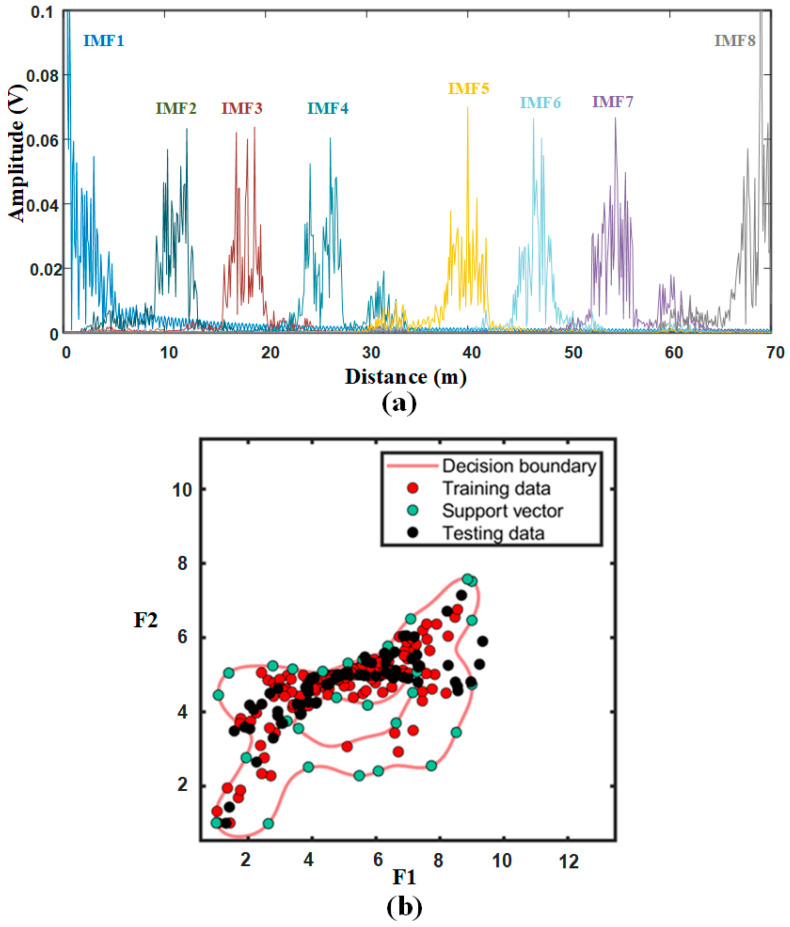
Signal processing for scene 1: (**a**) Mode components after VMD processing; (**b**) SVDD classification result.

Figure 14a shows mode components after VMD processing of scene 1. Where, IMF2, IMF3, IMF6 and IMF7 are selected as clutter modes to train the SVDD classifier, and target modes are IMF4 and IMF5. Through analysis of the measured data, the first IMF and the last IMF have a great influence on the final selection result. Therefore, both modes are discarded in the selection threshold estimation, which is the same as the simulation. According to the actual situation, the decomposition layer number K of VMD is adaptively adjusted to 8. The SVDD classification result is shown in Figure 14b. The sampling points outside the decision boundary belongs to the target or false alarm.

Here, both Figure 13b and Figure 14b show the detection result of single target. Figure 13b represents the result in the distance dimension, while Figure 14b represents the result in the feature space. For scene 1, the final detection result is that two false alarms appear around the single target, so there are three lines in Figure 13b, corresponding to three points outside the decision boundary in Figure 14b.

**Figure 15:**
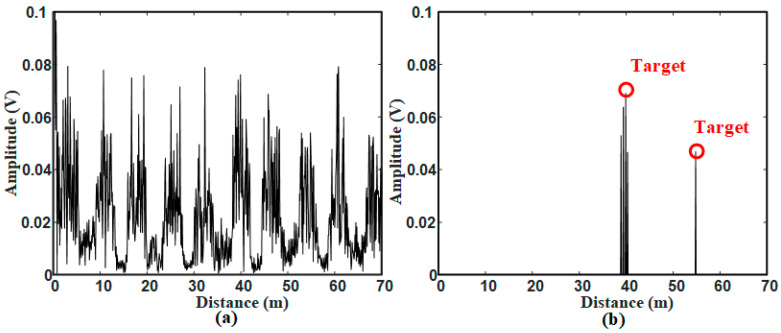
Signals in scene 2: (**a**) Original signal; (**b**) Target detection result.

For scene 2, the original signal is shown as Figure 15a,b shows the detection result that both targets are correctly detected. There are still false alarms around the target 40 m from the radar sensor.

In fact, if false alarm objects are very close to the target, it has little impact on the detection result. In the future work, how to improve the detecting probability of such situation will be further researched.

## 5. Conclusions

The FOD detection method in the MMW radar sensor system is limited by the strong ground clutter on the airport runway. This work presents an improved VMD-SVDD method to detect FODs. In this method, the echo signal received by MMW radar system are is firstly decomposed into BLIMFs with the optimal VMD. Then the mode components divided into two parts: FOD mode components and clutter components. The selected clutter mode components are used to train the SVDD classifier, and then the FOD mode components are tested by the classifier. This method relies on the SVDD classifier to distinguish FOD signals from clutter signals, what’s more, the accuracy and stability of the classifier is improved by the optimal VMD. The proposed method has two significant advantages:

(1) The VMD-SVDD method is more adaptable. There is no need to set VMD parameters, among which quadratic penalty parameter α and decomposition layer number K are searched by the WOA algorithm;
(2) The VMD-SVDD method can effectively suppress ground clutter signals and has the higher detection probability.

After analytically describing the procedure, the effectiveness of the approach has been proven by simulation. Furthermore, the general validity of the method is evidenced with the measured data.

## Figures and Tables

**Table 1:** Parameters of the LFMCW radar system.

Parameter	Value	Parameter	Value
bandwidth	1.5 GHz	antenna gain	20 dBi
sampling frequency	20 MHz	horizontal beam width	1.9°
farthest monitoring distance	70 m	pitch beam width	5°
range resolution	0.1 m	beam width in azimuth	120°
FFT point number	1024	beam width in downward	28°
frequency modulation cycle	128 us	angular step	12°/s
pulse accumulation number	468	cumulative time	60 ms

**Table 2:** Real-time analysis of four methods.

Method	CA-CFAR	VMD-CACFAR	SVDD	VMD-SVDD
**Run time (s)**	0.25	9.87	4.91	10.93

## Data Availability

The data presented in this study are available on request from the corresponding author. The data are not publicly available due to privacy.
